# Down‐regulation of exosomal miR‐200c derived from keratinocytes in vitiligo lesions suppresses melanogenesis

**DOI:** 10.1111/jcmm.15864

**Published:** 2020-09-11

**Authors:** Chaoshuai Zhao, Dongliang Wang, Xin Wang, Yaqi Mao, Ziqian Xu, Yue Sun, Xingyu Mei, Jun Song, Weimin Shi

**Affiliations:** ^1^ Department of Dermatology Shanghai General Hospital Shanghai Jiaotong University School of Medicine Shanghai China; ^2^ Department of Nuclear Medicine Renji Hospital Shanghai Jiaotong University School of Medicine Shanghai China

**Keywords:** exosomes, keratinocytes, melanogenesis, miR‐200c, vitiligo

## Abstract

Vitiligo is a refractory disfiguring skin disease. However, the aetiology and pathogenesis of vitiligo have not been fully defined. Previous studies have shown that exosomes from normal human keratinocytes improve melanogenesis by up‐regulating the expression of melanogenesis‐related proteins. Several microRNAs (miRNAs) have been demonstrated to be effective in modulating melanogenesis via exosomes. In the present study, it was found that the effect of exosomes derived from keratinocytes in vitiligo lesions in regulating melanin synthesis is weakened. Furthermore, miR‐200c was detected to be significantly down‐regulated in exosomes from keratinocytes in vitiligo lesions. In addition, miR‐200c enhanced the expression of melanogenesis‐related genes via suppressing SOX1 to activate β‐catenin. In conclusion, our study revealed that the effect of exosomes secreted by keratinocytes in vitiligo lesions exhibited a weaker capacity in promoting melanogenesis of melanocytes. Moreover, the expression of miR‐200c, which mediates melanogenesis in exosomes secreted by keratinocytes in vitiligo lesions, is down‐regulated, which may be one of the pathogenesis in vitiligo. Therefore, keratinocyte‐derived exosomal miR‐200c may be a potential target for the treatment of vitiligo.

## 1 INTRODUCTION

Vitiligo is an acquired disfiguring dermatosis characterized by patchy depigmentation due to the selective loss of functioning melanocytes. The incidence rate of vitiligo is approximately 0.5%‐1.0% of the world's population.^1^ At present, the aetiology of vitiligo remains unclear. Multiple studies demonstrated that massive oxidative stress may lead to vitiligo. Oxidation pathway is regarded as the important steps in the development of vitiligo.^2^ In human skin, each melanocyte forms an epidermal melanin unit with 36 keratinocytes, which play an important role in maintaining melanocyte homeostasis.^3^ Various growth factors produced by keratinocytes affect melanocyte proliferation and differentiation, such as endothelin‐1, stem cell factor and basic fibroblast growth factor.^4^, ^5^, ^6^ Additionally, keratinocyte‐derived soluble factors regulate the melanogenesis of neighbouring melanocytes.^7^ Moreover, various hormonal and peptide factors produced locally affect proliferation and activity of melanocytes. For example, UVB can stimulate CRH gene expression and peptide production in melanocytes which can affect epidermal proliferation of keratinocytes and melanocytes.^8^ Androgens and estrogens can regulate proliferation and melanogenesis in cultured melanocytes. Neuron growth factor (NGF) stimulates melanocyte dendrite formation and prolongs melanocytes survival after UV injury.^9^ L‐tyrosine and L‐dihydroxyphenylalanine (L‐DOPA), as substrates and intermediates of melanogenesis, not only induce but also positively regulate the process of melanogenesis.^10^ Some studies have demonstrated that keratinocyte‐derived factors are inadequate and that the ultrastructure of keratinocytes has changed in vitiligo. The alteration of keratinocytes may be the cause of the disappearance of melanocytes in vitiligo.^11^, ^12^, ^13^

Exosomes are nano‐sized extracellular vesicles that are released from almost all cell types in a physical or pathological state.^14^ These can mediate cell‐to‐cell communication by transferring proteins and various nucleic acids, including mRNAs, miRNAs and small regulatory RNAs (sRNAs), to neighbouring cells or distant cells.^15^, ^16^ Current studies have found that keratinocyte‐derived exosomes modulated melanogenesis in melanocytes.^17^, ^18^

MicroRNAs (miRNAs) are small endogenous non‐coding RNAs that play a key role in regulating gene expression at the post‐transcriptional level.^19^ It was identified that miR‐3196 in exosomes derived from keratinocytes enhanced the gene expression of microphthalmia‐associated transcription factor (MITF) in melanocytes. Mir‐203, in contrast to miR‐3196, increases the tyrosinase (TYR) expression, without modifying the gene expression of MITF.^17^ However, some miRNAs from keratinocyte exosomes negatively regulate melanogenesis in melanocytes, such as miR‐330‐5p and miR‐675.^20^, ^21^

In vitiligo, we investigated whether exosomes derived from keratinocytes still have the ability to modulate melanogenesis in melanocytes. Our study revealed that exosomes secreted by VLK have a weaker capacity for promoting melanogenesis of melanocytes. Furthermore, the down‐regulated expression of miR‐200c carried by exosomes from keratinocytes in vitiligo lesions to target melanocytes led to the decrease in tyrosinase activity and melanogenesis‐related gene expression.

## 2 MATERIALS AND METHODS

### 2.1 Sample collection, ethical statement, and cell culture

Normal human epidermal melanocytes (NHEM) and normal human epidermal keratinocytes (NHEK) were isolated from male adult foreskins obtained at circumcision. The epidermis was separated from the dermis and treated with 0.25% trypsin after digesting foreskins by 0.25% dispase at 4°C for 18 hours. Under a negative pressure of 250 mm Hg, suction blisters of depigmented skin in vitiligo were obtained, and keratinocytes in the vitiligo lesions (VLK) were further isolated. All participants provided a signed informed consent. The present study was approved by the Institutional Research Ethics Committee of Shanghai General Hospital and abided by the ethical guidelines of the Declaration of Helsinki. The keratinocytes were cultured in Keratinocytes Medium‐defined (#2111; ScienCell, San Diego, CA, USA). The melanocytes were grown in Medium 254 (M254500; Thermo Fisher Scientific, Carlsbad, CA, USA) supplemented with Human Melanocyte Growth Supplement‐2 (S0165, Thermo Fisher Scientific).

### 2.2 Exosome isolation

The exosomes were isolated from the conditioned medium of keratinocytes. Briefly, the supernatant was filtered through a 0.22‐μm syringe filter and centrifuged at 120 000 g for 90 minutes (4°C). Then, the pellet was washed with phosphate‐buffered saline (PBS) before another 90 minutes of ultracentrifugation at 120 000 g (4°C). In the last step, the pellet was resuspended and stored at −80°C. All steps of ultracentrifugation are performed using the Optima™ XPN‐100.

### 2.3 Immunofluorescence (IF)

The exosomes were labelled using green fluorescent membrane dye PKH67 (PKH67GL; Sigma‐Aldrich, St. Louis, MO, USA), according to the protocol. Cells were cultured in Medium 254 containing exosomal solution labelled PKH67 or negative control solution for 24 hours at 37°C in six‐well plates. Then, the slides were washed for three times with PBS, fixed with 4% formaldehyde for 20 minutes and washed for three times again with PBS. Afterwards, the cell nuclei were stained using DAPI fluorescent stain (D9542, Sigma‐Aldrich). The images were acquired using the ImageXpress Micro^®^ Confocal High‐Content Imaging System (Molecular Devices).

### 2.4 TYR activity assay

Melanocytes were treated with exosomes (25 μg/mL) obtained from keratinocytes for 72 hours, then digested in 0.25% trypsin and washing with PBS twice. Then, these cells were suspended in phosphate buffer containing 200 μL of 1% TritonX‐100 (X100, Sigma‐Aldrich). Afterwards, the cell lysate was collected after centrifugation at 13 000 g for 10 minutes at 4°C. Next, 0.1% L‐DOPA was added to dilute the sample to 1 mL, and 200 μL per well was added to the 96‐well plate and incubated at 37°C for 30 minutes. The absorbance value was measured by the enzyme mark instrument at 475 nm, and the tyrosinase activity (normalized to control) in melanocytes was calculated. Tyrosinase activity (normalized to control) = [(A_30_‐A_0_)/N]/[(A_C30_‐A_C0_)/N_C_]. A_0_ and A_30_ refer to the absorbance values of the VLK group or the NHEK group in 0 and 30 minutes. A_C0_ and A_C30_ refer to the absorbance values of the control group in 0 and 30 minutes. N refers to the number of melanocytes in the VLK group or the NHEK group. N_C_ refers to the number of melanocytes in the control group.^22^

### 2.5 Melanin content assay

After the melanocytes were treated with exosomes (25 μg/mL) for 72 hours, these were digested with 0.25% trypsin and washed twice with PBS. Then, the cells were dissolved in 1 mol/L NaOH at 80°C for 30 minutes, and the melanin content was measured as OD at 475 nm.

### 2.6 Quantitative real‐time PCR

The total RNA of the control and treated cells or exosomes was isolated using the RNeasy Mini kit (Qiagen, Hilden, Germany), the total RNA of exosomes was isolated using the exoRNeasy Midi Kit (Qiagen, Hilden, Germany), and the cDNA was synthesized using HiScript II Q RT SuperMix for qRT‐PCR (R223‐01, Vazyme). Quantitative real‐time PCR for mRNA and miRNA expression analysis was carried out using QuantStudio^TM^ 7 Flex (Thermo Fisher Scientific), and this was normalized using β‐actin (for mRNA) or RNU6 (for miRNA). The data were analysed using the delta Ct method. The primers used for the present study are listed in Table 1.

**Table 1:** Primers used in this study

Primer	Sequences
TYR Forward primer	AGCCTGTGCCTCCTCTAA
TYR Reverse primer	AGGAACCTCTGCCTGAAA
MITF Forward primer	TCTACCGTCTCTCACTGGATTGG
MITF Reverse primer	GCTTTACCTGCTGCCGTTGG
TRP1 Forward primer	TCTCTGGGCTGTATCTTCTTCC
TRP1 Reverse primer	GTCTGGGCAACACATACCACT
TRP2 Forward primer	AACTGCGAGCGGAAGAAACC
TRP2 Reverse primer	CGTAGTCGGGGTGTACTCTCT
miR‐200c Forward primer	GGGAACACACCTGGTTAAC
miR‐200c Reverse primer	CAGTGCGTGTCGTGGAGT
miR‐200a‐3p Forward primer	GCGCGTGAAATGTTTAGGAC
miR‐200a‐3p Reverse primer	GTGCAGGGTCCGAGGT
miR‐1246 Forward primer	TGAAGTAGGACTGGGCAGAGA
miR‐1246 Reverse primer	TGTTTGCAATAGCCCTTTGAG
β‐actin Forward primer	CATCCTCACCCTGAAGTACCCC
β‐actin Reverse primer	AGCCTGGATGCAACGTACATG
U6 Forward primer	CGCTTCGGCAGCACATATAC
U6 Reverse primer	TTCACGAATTTGCGTGTCAT

### 2.7 MiRNA transfection

The miR‐200c mimics, miR‐200c inhibitor and negative control (NC) were obtained from GenePharma (Shanghai, China). The melanocytes and keratinocytes were transfected using the Lipofectamine™ 3000 Transfection Reagent (L300001, Invitrogen, Carlsbad, CA, USA) for 48 hours. Then, the exosomes were isolated from the conditioned medium after 48 hours.

### 2.8 Western blot analysis

Cells were lysed in RIPA buffer (P0013C, Beyotime, Shanghai, China) with a protease inhibitor cocktail (P8340, Sigma‐Aldrich). The total proteins of lysates or exosomes were separated by SDS polyacrylamide gels and transferred onto PVDF membranes (Millipore, USA). Then, these membranes were blocked with 5% BSA for one hour at room temperature and incubated with primary antibodies overnight at 4°C. Afterwards, the secondary antibodies were added. The blots were observed using the Azure Biosystems C300. The antibodies are listed, as follows: rabbit anti‐Tyrosinase (ab170905; Abcam, Cambridge, MA, USA), rabbit anti‐TRP1 (ab178676, Abcam), rabbit anti‐TRP2/DCT (ab221144, Abcam), rabbit anti‐MITF (ab140606, Abcam), rabbit anti‐β‐catenin (51067‐2‐AP; Proteintech, Wuhan, China), rabbit anti‐SOX1 (20744‐1‐AP, Proteintech), rabbit anti‐CD63 (25682‐1‐AP, Proteintech), rabbit anti‐TSG101 (14497‐1‐AP, Proteintech), Lamin B1 antibody (12987‐1‐AP, Proteintech) and β‐actin (no.3700, Cell Signaling Technology, Danvers, MA, USA).

### 2.9 MiRNA sequencing

The total RNAs were extracted from exosomes isolated from the conditioned medium of keratinocytes. The miRNA library preparation and sequencing were performed by a commercial service (Ribobio, China). The 3′ and 5′ splices were successively connected. Then, the reverse transcription was carried out to the cDNA, followed by PCR amplification. The cDNA library was sequenced using the Illumina HiSeq 2500 platform.

### 2.10 Transmission electron microscopy (TEM)

Exosomes resuspended in PBS were fixed with 4%PFA (at 1:1 dilution) and deposited on formvar‐carbon‐coated grids. Then, these copper grids were fixed with 1% glutaraldehyde for 5 minutes and washed with ddH_2_O for eight times after washing the PBS droplets. Afterwards, negative staining with uranium acetate was performed for 10 minutes, followed by absorption of the excess liquid with a filter paper. The samples were observed using a transmission electron microscopy at 80 kV.

### 2.11 Nanoparticle tracking analysis (NTA)

Nanoparticle tracking analysis (NTA) was used to analyse the size distribution of exosomes through the ZetaView PMX 110 (Particle Metrix, Meerbusch, Germany). The exosomal samples were diluted to a concentration of 1 × 10^8^ particles/mL with filtered PBS. The moving particles were recorded in video for five times, for 30 seconds each. Then, the data were analysed using the ZetaView 8.04.02 software.

### 2.12 Luciferase assay

The sequences for the SOX1 3’ UTR that contained the potential wide‐type or mutant binding sites of miR‐200c were constructed into pmirGLO vectors (Promega). The miR‐200c and luciferase vectors were co‐transfected in melanocytes, along with the pRL‐TK vector, by Lipofectamine 3000 (Invitrogen). The preparation of mutant reporter plasmids was performed using a Mutagenesis Kit (Stratagene, La Jolla, CA, USA). The Dual‐Luciferase Reporter Assay Kit (Promega, Madison, WI, USA) was used to detect the luciferase activity of these samples.

### 2.13 Statistical analysis

All data were expressed as mean ± standard deviation (SD). One‐way ANOVA was used to analyse the statistical significance of the difference in three or more than three groups, and t test was used to calculate the statistical difference between the two groups. The statistical analysis was performed by the SPSS program (version 13.0; SPSS, Chicago, IL, USA) and GraphPad Prism 5 (GraphPad, San Diego, CA, USA). *P* < 0.05 was considered statistically significant.

## 3 RESULTS

### 3.1 Exosomes secreted by VLK exhibit a weaker capacity in promoting melanogenesis of melanocytes than those secreted by NHEK

**Figure 1:**
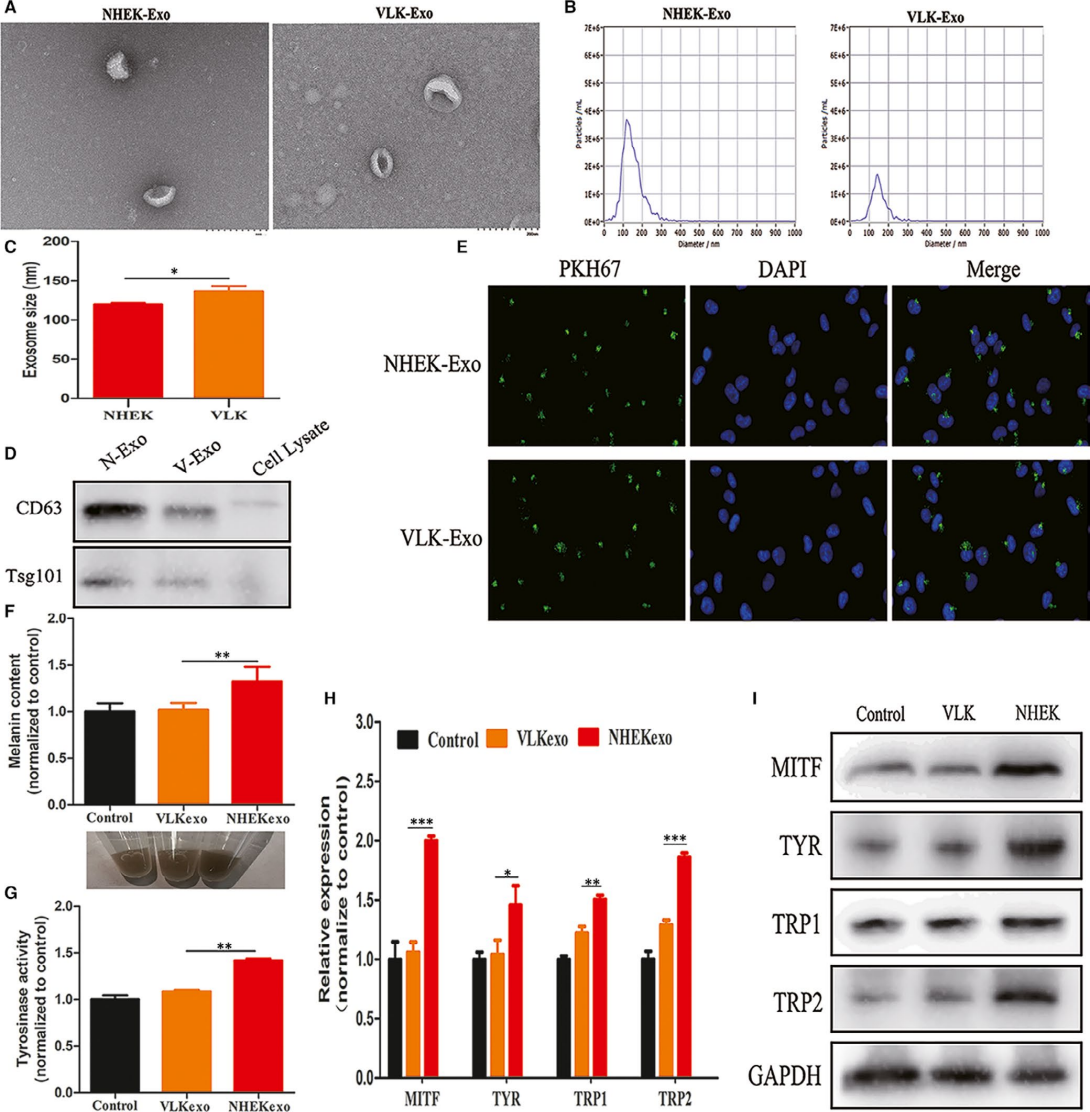
Exosomes characterization, uptake and their effects on the regulation of melanogenesis. A, The morphology of NHEK‐Exo and VLK‐Exo observed by transmission electron microscope (TEM) (Scale bars, 200 nm). B, Exosomes size distribution measured by nanoparticle tracking analysis (NTA). C, Statistical analysis of exosome size between NHEK and VLK (*P* < 0.05). D, Western blot analysis of exosome specific surface markers (CD63, TSG101) in NHEL‐Exo, VLK‐Exo and cell lysate. E, IFM analysis of the interaction of PKH67‐labelled (green) exosomes from NHEK or VLK with melanocytes labelled for DAPI (blue). (F and G) Melanin content, cell pellets and tyrosinase activity in melanocytes cocultured with NHEK‐Exo or VLK‐Exo for 72 h. Melanocytes cocultured with PBS were used as a control. (H and I) Analysis by qRT‐PCR and Western blot on the expression of MITF, TYR, TRP1 and TRP2 in melanocytes cocultured with NHEK‐Exo or VLK‐Exo for 72 h. Melanocytes cocultured with PBS were used as a control. Results are expressed as mean ± SD (n = 3). *P < 0.05, **P < 0.01, ***P < 0.005

To characterize the exosomes derived from NHEK and VLK, ultracentrifugation was applied to isolate exosomes from the culture supernatants of keratinocytes. Based on TEM, vesicles that contained the typical cup‐shaped structure of exosomes were observed in both healthy and patient samples (Figure 1A). NTA showed that the average diameter of exosomes from NHEK was 120 ± 1.3 nm and that of exosomes from VLK was 136.2 ± 4.0 nm (Figure 1B). The exosome size was slightly larger in keratinocytes from vitiligo lesions than keratinocytes from normal human epidermis (*P* < 0.05) (Figure 1C). Exosomal protein markers, including CD63 and TSG101, were identified in exosomes from both NHEK and VK by Western blot (Figure 1D).

It has been reported that exosomes released by normal keratinocytes can modulate melanocyte pigmentation. In order to compare the capacity of exosomes secreted by NHEK and VLK in promoting the melanogenesis of melanocytes, exosomes from both NHEK and VK were added into melanocytes obtained from healthy controls (HCs). Initially, through the immunofluorescence assay performed with PKH‐67‐labelled exosomes, the ability of exosomes to enter the melanocytes was identified (Figure 1E). Then, it was observed that the intracellular melanin content and TYR activity of melanocytes incubated with patient exosomes were significantly lower than those of melanocytes incubated with NHEK exosomes (Figure 1F,G). Accordingly, melanocytes treated with exosomes from VLK presented with lower mRNA and protein levels of MITF, TYR, TRP1 and TRP2, when compared to those treated with exosomes from NHEK (Figure 1H,I). Taken together, these data suggest that exosomes secreted by VLK exhibit a weaker capacity in promoting the melanogenesis of melanocytes than those secreted by NHEK.

### 3.2 MiR‐200c is down‐regulated in exosomes from VLK

**Figure 2:**
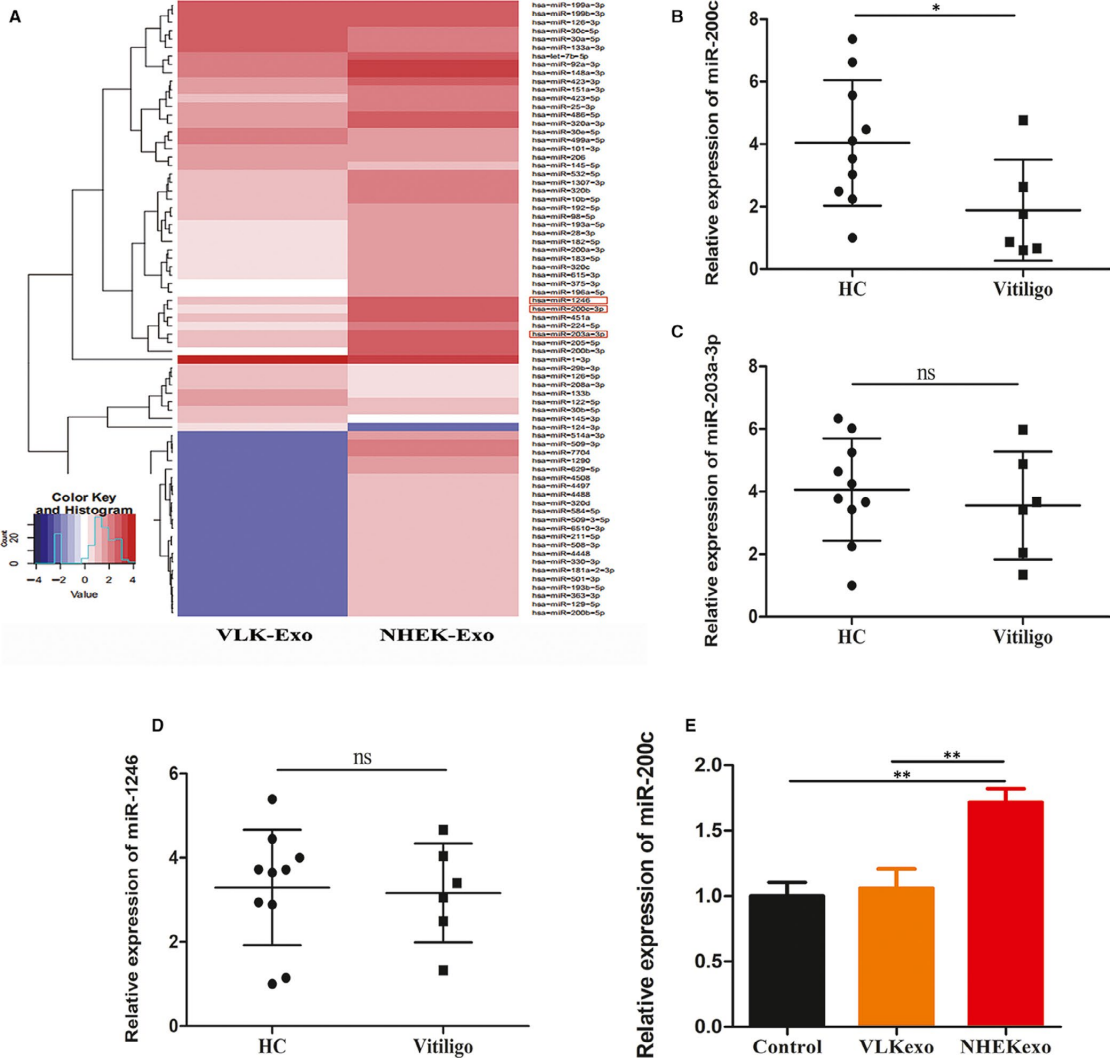
Expression levels of miR‐200c in exosomes from NHEK and VLK. A, Heat map of miRNA expression profiles of NHEK exosomes and VLK exosomes. Red or blue colour in heat map respectively indicates high or low expression. (B, C and D) qRT‐PCR analysis of the exosomal miR‐200c, miR‐203a and miR‐1246 expression levels in patients (n = 6) and healthy controls (n = 10). E, qRT‐PCR analysis of the miR‐200c levels in melanocytes treated with exosomes from NHEK and VLK for 72 h. Results are expressed as mean ± SD. *P < 0.05, **P < 0.01, ***P < 0.005

Some studies have reported that exosomal miRNAs derived from keratinocytes modulate pigmentation in melanocytes. To explore whether exosomal miRNAs participate in the modulation of melanogenesis in melanocytes, the miRNA profile of VLK and NHEK exosomes was analysed. In the present study, 73 differentially expressed miRNAs (20 up‐regulated and 53 down‐regulated miRNAs) were identified (Figure 2A). Then, the qualified miRNA was further selected using the following criteria: (a) log_2_ fold change of more than 3, and *P* < 0.001; (b) miRNA marked as ‘detected’ in at least one group of all samples. A total of 14 miRNAs (3 up‐regulated and 11 down‐regulated miRNAs) were selected. Subsequently, the qRT‐PCR assays were used to validate the expression of the top three miRNAs with the highest fold changes in exosomes from 10 NHEK samples and 6 VLK samples. However, merely miR‐200c was verified to be anomalously expressed in exosomes from VLK, when compared with those from NHEK (Figure 2B‐D). Furthermore, it was exhibited that the melanocytes incubated with exosomes from VLK had a lower miR‐200c level compared with melanocytes treated with exosomes from NHEK (Figure 2E). Therefore, miR‐200c was chosen to further investigate its role in melanocytes.

### 3.3 MiR‐200c facilitates melanogenesis of melanocytes

**Figure 3:**
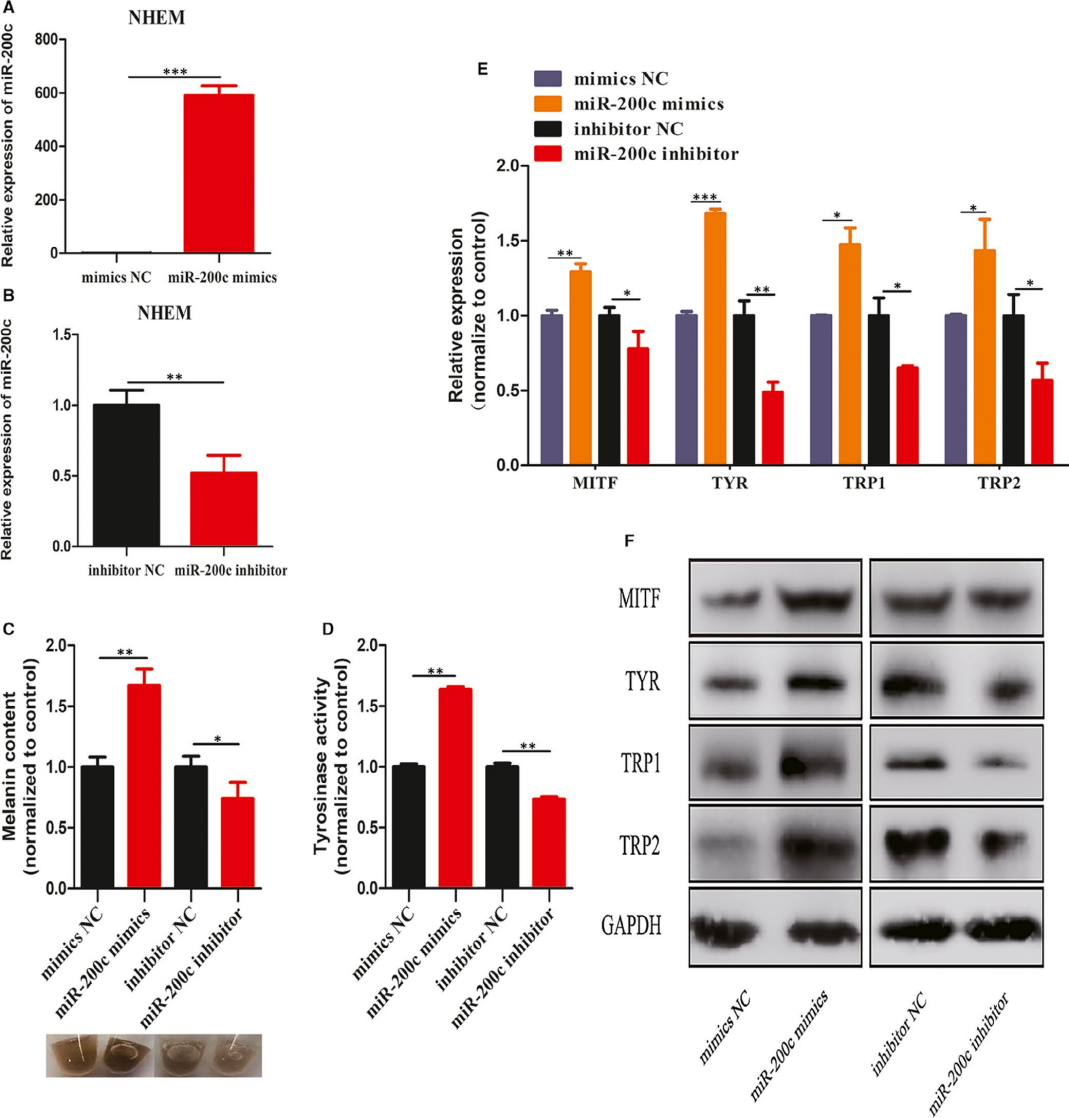
Effects of miR‐200c on melanocyte pigmentation. (A and B) Melanocytes were transfected with miR‐200c mimics or miR‐200c inhibitor for 48 h. (C and D) Intracellular melanin content, cell pellets and tyrosinase activity analysis in melanocytes transfected with miR‐200c mimics or miR‐200c inhibitor. (E and F) qRT‐PCR and Western blot were used to analyse the expression of MITF, TYR, TRP1 and TRP2 in melanocytes transfected with miR‐200c mimics or miR‐200c inhibitor on mRNA and protein level, respectively. Results are expressed as mean ± SD (n = 3). *P < 0.05, **P < 0.01, ***P < 0.005

To explore the functions of miR‐200c in modulating melanogenesis of melanocytes, this was overexpressed by the transfection of miR‐200c mimics and knocked down by the transfection of miR‐200c inhibitor (Figure 3A,B). Then, the intracellular melanin content and TYR activity of melanocytes, as well as the mRNA and protein levels of MITF, TYR, TRP1 and TRP2, were measured. These results demonstrate that the overexpression of miR‐200c significantly increased the intracellular melanin content, TYR activity, and expression of MITF, TYR, TRP1 and TRP2 (Figure 3C‐F). Accordingly, knock‐down of miR‐200c decreased the intracellular melanin content, TYR activity and expression of MITF, TYR, TRP1 and TRP2 (Figure 3C‐F). These results revealed that miR‐200c facilitate melanogenesis of melanocytes.

### 3.4 Exosomes secreted by keratinocytes regulate melanogenesis of melanocytes via miR‐200c

**Figure 4:**
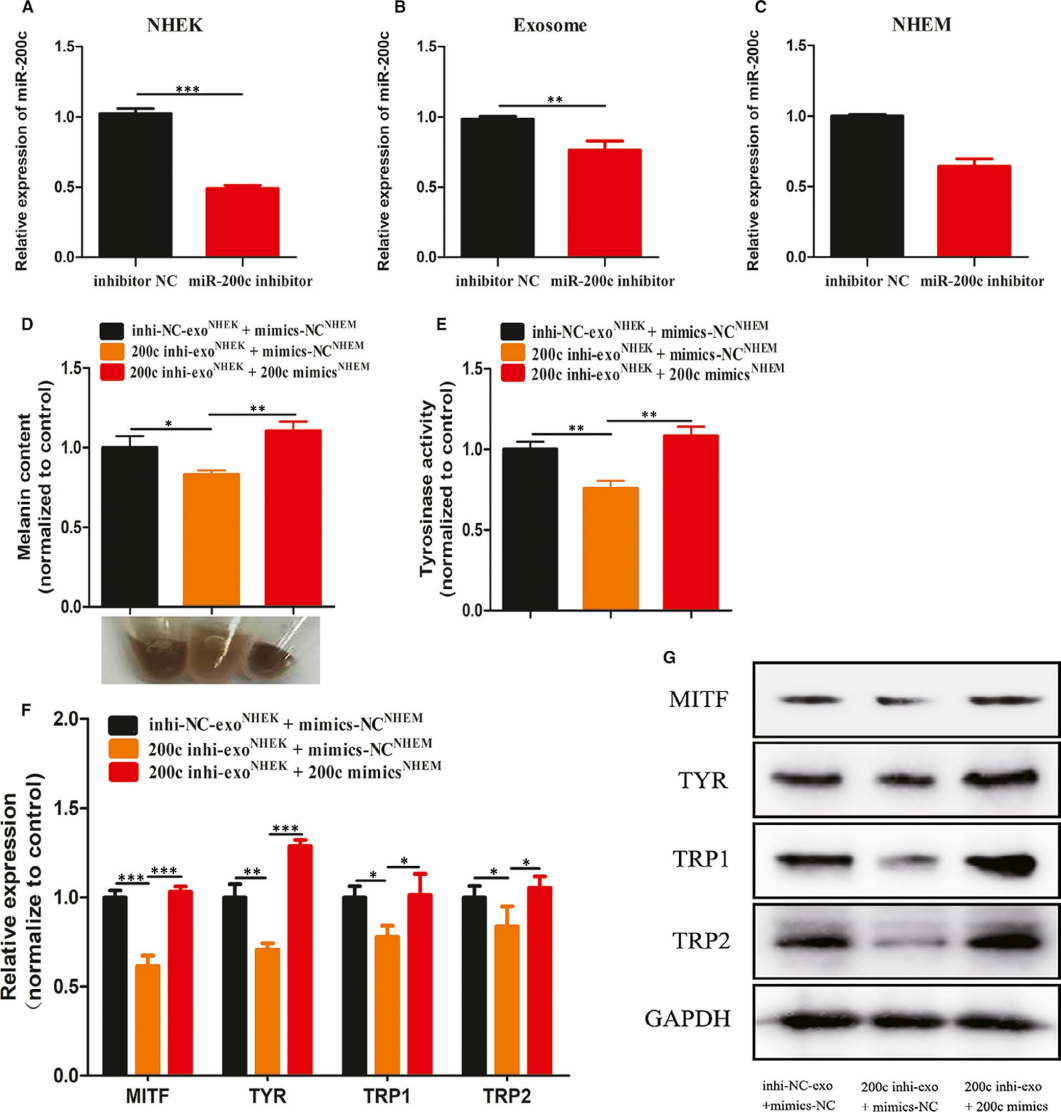
Exosomes modulate melanin synthesis via miR‐200c. A, The expression level of miR‐200c in keratinocytes transfected with miR‐200c inhibitor. B, The expression level of miR‐200c in exosomes secreted by keratinocytes which down‐regulated miR‐200c. C, MiR‐200c expression level in exosome‐treated melanocytes with down‐regulated Mir‐200c expression. (D and E) Intracellular melanin content, cell pellets and tyrosinase activity in melanocytes treated with corresponding exosomes and plasmids. melanocytes incubated with exosomes from keratinocytes transfected with miR‐200c mimics or miR‐NC. (F and G) qRT‐PCR and Western blot were used to analyse the expression of MITF, TYR, TRP1 and TRP2 in melanocytes treated with corresponding exosomes and plasmids. Results are expressed as mean ± SD (n = 3). *P < 0.05, **P < 0.01, ***P < 0.005

From these above results, it can be observed that both exosomes that lacked the miR‐200c and transfected with miR‐200c inhibitor exerted similar functions in being insufficient to maintain the melanogenesis in melanocytes. Consequently, we attempted to determine whether miR‐200c was the key player through which exosomes modulated melanogenesis of melanocytes. Hence, the miR‐200c inhibitor was transfected into keratinocytes and isolated exosomes from keratinocytes to treat melanocytes. In the present study, it was found the miR‐200c levels decreased in keratinocytes transfected with miR‐200c inhibitor, the exosomes secreted by keratinocytes, and the melanocytes treated with exosomes (Figure 4A‐C). Furthermore, it was also found that melanocytes treated with exosomes of miR‐200 knocked down presented similar characteristics in melanogenesis with melanocytes treated with exosomes from VLK (Figure 4D‐G). More importantly, when miR‐200c was recuperated by the transfection of miR‐200c mimics in melanocytes treated with exosomes of miR‐200c knocked down, the capability of melanocytes to maintain the melanogenesis recovered (Figure 4D‐G). All the above results illustrate that exosomes secreted by keratinocytes regulate melanogenesis of melanocytes via miR‐200c.

### 3.5 Exosomes secreted by keratinocytes regulate the activation of β‐catenin in melanocytes by mediating SOX1 via miR‐200c

**Figure 5:**
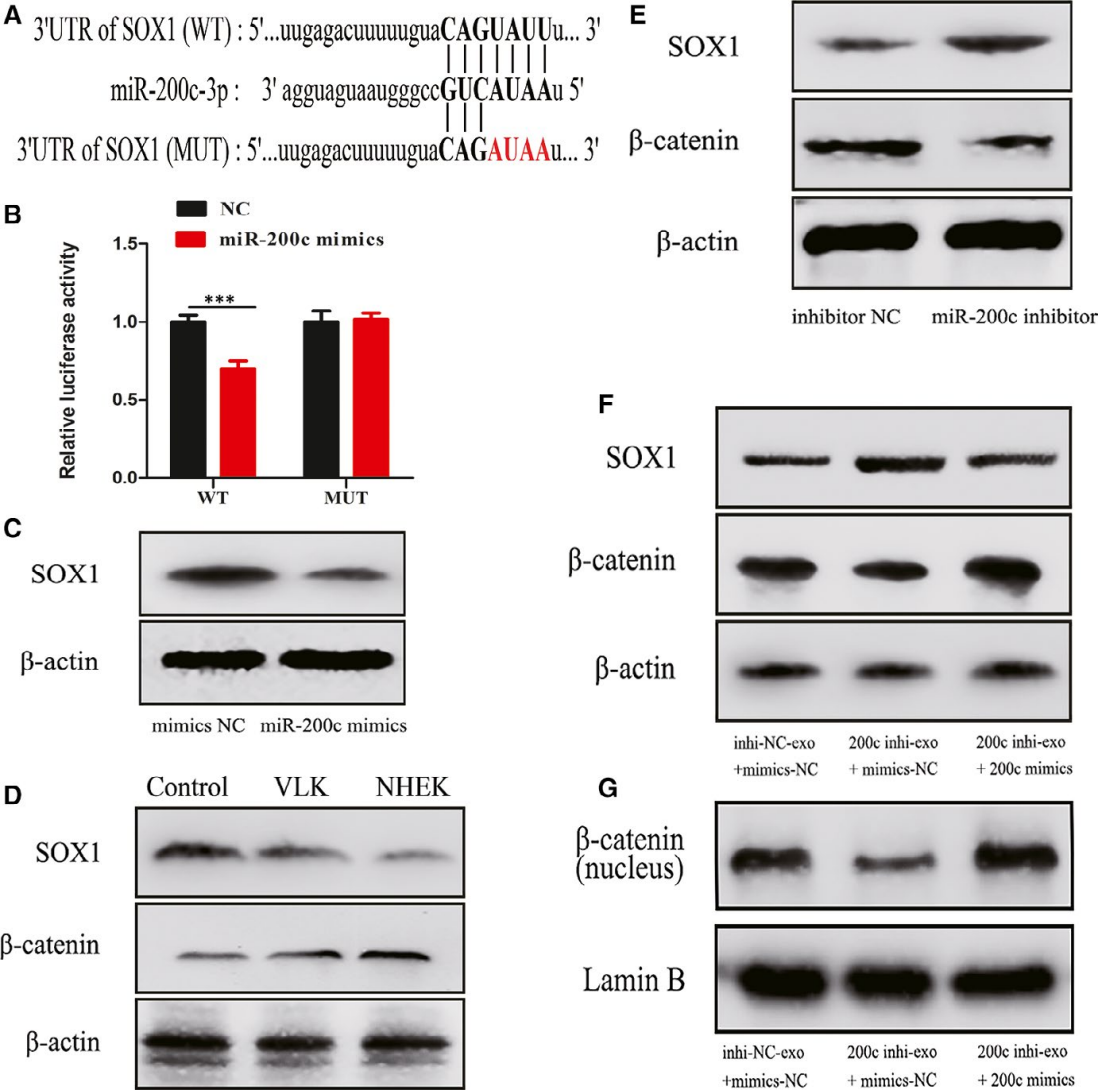
Exosomal miR‐200c regulates the activation of β‐catenin by targeting SOX1. A, Predicted binding sites and corresponding mutant sites in the 3' UTR of SOX1 mRNA (WT, wild‐type; MUT, mutant type). B, Relative SOX1 reporter activity in melanocytes co‐transfected with miR‐200c and luciferase reporters. C, Western blot analysis of SOX1 protein expression in melanocytes transfected with miR‐200c mimics. D, SOX1 and β‐catenin protein levels in melanocytes cocultured with NHEK or VLK exosomes for 72 h. E, Western blot analysis of SOX1 and β‐catenin expression in melanocytes transfected with miR‐200c inhibitor. F, Western blot analysis of SOX1, β‐catenin expression in melanocytes treated with corresponding exosomes and plasmids. G, Western blot analysis of the nuclear‐β‐catenin expression in melanocytes treated with corresponding exosomes and plasmids. Results are expressed as mean ± SD (n = 3). *P < 0.05, **P < 0.01, ***P < 0.005

To illustrate the mechanism of exosomes/miR‐200c in regulating melanogenesis, we sought the target genes of miR‐200c. SOX1, which was identified by miRDB, Targetscan and microRNA.org databases, was selected as a potential candidate. To verify the direct binding between the SOX1 3’UTR and miR‐200c, wild‐type (wt) and mutant (mut) reporter gene plasmids containing the putative binding site of SOX1 were constructed (Figure 5A). The luciferase reporter assays revealed that miR‐200c suppressed the luciferase activity in melanocytes of the reporter with the wild‐type SOX1 3’ UTR, but not of the reporter with the mutant‐type SOX1 3’ UTR (Figure 5B). In addition, the SOX1 protein expression was significantly inhibited when melanocytes were transfected with miR‐200c mimics (Figure 5C). Hence, it was concluded that SOX1 is the target gene of miR‐200c, which can be suppressed by miR‐200c.

**Figure 6:**
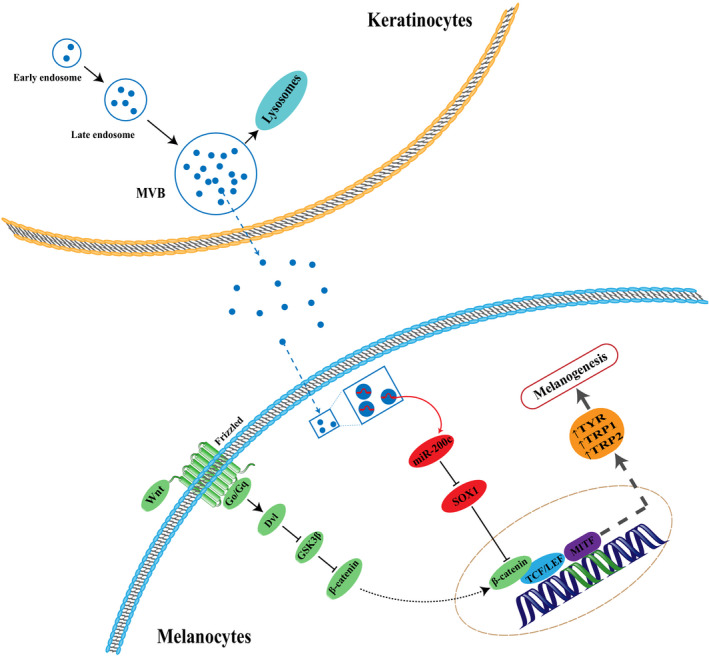
A model illustrating the role of exosomal miR‐200c derived from keratinocytes in regulating melanogenesis of melanocytes

What's more, when melanocytes were treated with exosomes from VLK, the miR‐200c inhibitor or exosomes of miR‐200 knocked down, these exhibited a relatively higher SOX1 expression, when compared with the respective NC groups (Figure 5D‐F). When miR‐200c was recuperated in melanocytes treated with exosomes of miR‐200 knocked down, the expression of SOX1 recovered (Figure 5F). SOX1 has been demonstrated to down‐regulate β‐catenin and reverse the malignant phenotype in nasopharyngeal carcinoma.^23^ Here, we uncovered that exosomes/miR‐200c could thereby modulate the expression of β‐catenin and the nuclear level of β‐catenin via SOX1 (Figure 5F,G). Thus, these present results indicate that exosomes secreted by keratinocytes regulate the activation of β‐catenin in melanocytes by mediating SOX1 via miR‐200c (Figure 6).

## 4 DISCUSSION

The process of delivering melanin from melanocytes to keratinocytes is very critical for skin protection against UVB and pigmentation.^24^, ^25^ UVB can up‐regulate the expression and activity of the α‐MSH receptor, the expression of POMC peptide production including that of α‐MSH, β‐endorphin and ACTH, to regulate skin pigmentation, to protect skin from UV induced injury, and to modulate skin immune response.^26^ The decrease in melanocytes and the dysfunction of melanogenesis are two distinct characteristics of vitiligo. However, in vitiligo, both the impairment of melanocytes and the morphology and function of keratinocytes exist. The interaction between keratinocytes and melanocytes plays an important role in maintaining the homeostasis of the epidermis.^27^ Compared with normally pigmented epidermis, the apoptosis of keratinocytes is more likely to occur in depigmented suction‐blistered epidermis, which can decrease the levels of keratinocyte‐derived factors, such as SCF and basic fibroblast growth factor. The reduction in these survival factors causes the apoptosis of melanocytes.^11^ Cicero *et al* reported that exosomes from black and irradiated Caucasian NHEK can up‐regulate the melanogenesis of melanocytes, while exosomes from non‐irradiated Caucasian NHEK did not. In addition, the miRNA profiles of keratinocyte exosomes in black and irradiated Caucasians differ from those of non‐irradiated Caucasians.^17^ On this basis, we further determined whether the function of modulating melanogenesis in melanocytes by exosomes derived from keratinocytes in vitiligo lesions changed.

Exosomes are small intracellular vesicles with a diameter of 40‐150 nm.^28^ In the present study, the size of the exosomes of VLK was slightly larger than that of NHEK, although TEM and NTA revealed that both of them were within the size range indicative of exosomes. Singh *et al* reported larger keratinocytes in vitiligo lesional skin, when compared to the non‐lesional stratum corneum.^12^ Changes in cell size are commonly observed in many disease conditions and during ageing.^29^ Senescence in human cells is correlated to the increase in cell size,^30^, ^31^ which influences the structural integrity of cells, increases intracellular distances and reduces surface‐to‐volume (SV) ratio, leading to metabolic inefficiency and cell death.^29^ In our study, the larger size of exosomes from VLK still needs to be confirmed through large sample studies, and further studies are needed to determine whether this is associated with the dysfunction in promoting melanogenesis.

Some studies have demonstrated that exosomes derived from keratinocytes modulate melanocyte melanogenesis. However, no study has determined whether the exosomes secreted by keratinocytes in vitiligo lesional skin still have the function in modulating melanogenesis. During our investigation, exosomes from NHEK promoted melanogenesis by up‐regulating the expression of MITF, TYR, TRP1 and TRP2, which was consistent with previous studies.^17^, ^18^ Compared with NHEK exosomes, the effect of exosomes from VLK in promoting melanin production was significantly reduced. In addition, it was observed that VLK exosomes still had a slight effect in promoting pigmentation compared with the control group. The present study showed that the effect of VLK exosomes in promoting melanogenesis was weakened. However, we did not find any evidence that VLK exosomes inhibit melanogenesis in melanocytes. Moreover, further studies are needed to analyse the processing of these melanogenesis‐related proteins because of double bands showed in Western blot, which may represent glycosylation.

In recent years, miR‐200c has been found to have two main functions in a variety of cancers, including melanoma. One function is to suppress the drug resistance of tumour cells,^32^, ^33^, ^34^ while the other function is to inhibit the invasion and migration of malignant cells.^35^, ^36^, ^37^ Cui *et al* implied that miR‐200c can suppress the chemoresistance of docetaxel in tongue squamous cell carcinoma through exosomes.^38^ The roles of miR‐200c have also been identified in many non‐malignant diseases and skin diseases. Liang et al showed that exogenous overexpression of miR‐200c inhibited the proliferation and migration of primary endometrial stromal cells.^39^ Previous studies have also demonstrated that miR‐200c can prevent the arecoline‐induced myofibroblast activator.^40^ Moreover, miR‐200c is the biomarker of atherosclerotic plaque progression, which may be useful in identifying patients at high embolic risk.^41^ Some studies showed that the inhibition of miR‐200c relieved the acute lung injury induced by H5N1 virus infection in vivo.^42^ In skin diseases, Magenta et al implicated miR‐200c was up‐regulated in psoriasis, which was related to the disease severity.^43^ Hence, we further explore the importance of miR‐200c in vitiligo. One of the most important findings in the present study was that miR‐200c was lowly expressed in exosomes from VLK compared with exosomes from NHEK. Moreover, we initially found that keratinocyte‐derived exosomal miR‐200c could stimulate melanin synthesis by up‐regulating the gene expression levels of MITF, TYR, TRP1 and TRP2. These results showed that the down‐regulation of miR‐200c in exosomes from keratinocytes in vitiligo lesions might affect the melanocyte melanogenesis in vitiligo.

SOX1 is a member of the sex‐determining region Y‐box (SOX) family, which encodes a transcription factor with a highly conserved high‐mobility group (HMG) DNA‐binding domain.^44^ Previous reports have revealed that SOX5, SOX6, SOX9, SOX10 and SOX18 are all involved in the regulation of key melanocytic genes as transcription factors.^45^, ^46^, ^47^, ^48^ In particular, SOX9 has been demonstrated to play a key role in melanogenesis in adults, and SOX10 has been found to seriously influence the development of melanocytes. Additionally, the link between SOX1 and β‐catenin has been shown in various carcinomas. It has been reported that SOX1 interacts with β‐catenin and induces proteasome‐independent down‐regulation of β‐catenin.^23^ In our study, the protein expression levels of total and intranuclear β‐catenin were up‐regulated with the down‐regulation of SOX1 expression, which was consistent with previous study. Our results indicate that exosomal miR‐200c derived from keratinocytes regulate the activation of β‐catenin in melanocytes by inhibiting SOX1.

The relationship between β‐catenin and MITF has been shown in several studies. MITF is a downstream target of Wnt signalling in the development of melanocytes.^49^ Schepsky *et al* implied that β‐catenin not only regulates the expression of MITF at the transcription level, but also directly interplays with MITF. Overexpression of β‐catenin improves MITF‐dependent gene expression.^50^ In this study, we found that β‐catenin up‐regulated the gene expression of MITF and in turn, promoted the expression of TYR, TRP1 and TRP2.

In general, the present study is the first to provide evidence that exosomes secreted by VLK have a weaker capacity in regulating melanogenesis of melanocyte, when compared to those secreted by NHEK, because the deficiency in miR‐200c expression affects the melanin synthesis. Our findings provide a new understanding of vitiligo pathogenesis, and further deeply illustrate the relationship between keratinocytes and melanocytes. In addition, keratinocyte‐derived exosomal miR‐200c is a promising target for vitiligo therapy. It may be more meaningful to analyse the effect of exosomes on melanocytes harvested from vitiligo patients. However, it is difficult to extract melanocytes from vitiligo lesions because of few melanocytes. Moreover, melanocytes in non‐lesional sites of vitiligo patients may still be in a normal state and can not reflect the situation of melanocytes in vitiligo lesions. So, we just analysed the effect of exosomes on melanocytes from normal human. Therefore, further studies are needed for its clinical treatment.

## CONFLICT OF INTEREST

The authors declare that there are no competing interests associated with the manuscript.

## AUTHOR CONTRIBUTION

**Chaoshuai Zhao:** Investigation (equal); Methodology (lead); Resources (equal); Validation (equal); Writing‐original draft (lead). **Dongliang Wang:** Investigation (equal); Methodology (equal); Validation (equal); Writing‐original draft (supporting). **Xin Wang:** Methodology (equal); Writing‐original draft (supporting). **Yaqi Mao:** Formal analysis (equal). **Ziqian Xu:** Formal analysis (equal). **Yue Sun:** Resources (equal). **Xingyu Mei:** Methodology (supporting); Resources (supporting). **Jun Song:** Supervision (equal); Writing‐review & editing (equal). **Weimin Shi:** Methodology (equal); Supervision (equal); Writing‐review & editing (equal).
